# DNA barcoding combined with high-resolution melting analysis to discriminate rhubarb species and its traditional Chinese patent medicines

**DOI:** 10.3389/fphar.2024.1371890

**Published:** 2024-06-14

**Authors:** Luyi Shen, Min Zhang, Yanmei Qiu, Lin Yang, Yiwen Lu, Hua Li, Leilei Zhang, Fan Tang, Feijuan Wang, Cheng Zhu, Hexigeduleng Bao, Yanfei Ding

**Affiliations:** ^1^ Key Laboratory of Specialty Agri-product Quality and Hazard Controlling Technology of Zhejiang Province, College of Life Science, China Jiliang University, Hangzhou, China; ^2^ Animal Disease Prevention and Control Centre, Bureau of Agriculture and Animal Husbandry and Science and Technology of Seda County, Seda, China; ^3^ Chengdu Agricultural College, Chengdu, China; ^4^ College of Engineering, Nanjing Agricultural University, Nanjing, China

**Keywords:** rhubarb, DNA barcoding, ITS2, HRM, traditional Chinese patent medicine

## Abstract

**Introduction:** Rhubarb is a frequently used and beneficial traditional Chinese medicine. Wild resources of these plants are constantly being depleted, meaning that rhubarb products have been subjected to an unparalleled level of adulteration. Consequentially, reliable technology is urgently required to verify the authenticity of rhubarb raw materials and commercial botanical drugs.

**Methods:** In this study, the barcode-DNA high-resolution melting (Bar-HRM) method was applied to characterize 63 rhubarb samples (five Polygonaceae species: *Rheum tanguticum*, *Rh. palmatum*, *Rh. officinale*, *Rumex japonicus *and* Ru.* sp.) and distinguish the rhubarb contents of 24 traditional Chinese patent medicine (TCPM) samples. Three markers, namely ITS2, *rbcL* and* psbA-trnH*, were tested to assess the candidate DNA barcodes for their effectiveness in distinguishing rhubarb from its adulterants. A segment from ITS2 was selected as the most suitable mini-barcode to identify the botanical drug rhubarb in TCPMs. Then, rhubarbs and TCPM samples were subjected to HRM analysis based on the ITS2 barcode.

**Results:** Among the tested barcoding loci, ITS2 displayed abundant sites of variation and was effective in identifying Polygonaceae species and their botanical origins. HRM analysis based on the ITS2 mini-barcode region successfully distinguished the authenticity of five Polygonaceae species and eight batches of TCPMs. Of the 18 TCPM samples, 66.7 % (12 samples) were identified as containing *Rh. tanguticum* or *Rh. officinale*. However, 33.3 % were shown to consist of adulterants.

**Conclusions:** These results demonstrated that DNA barcoding combined with HRM is a specific, suitable and powerful approach for identifying rhubarb species and TCPMs, which is crucial to guaranteeing the security of medicinal plants being traded internationally.

## Introduction

*Rheum* (Polygonaceae), commonly known as rhubarb, or Radix et Rhizoma Rhei, grows widely in Asia’s high mountainous areas under temperate and subtropical climates (Zhou et al., 2020). The Shennong Herbal Classic, written in 270 BC, contains the first recorded uses of rhubarb as a traditional Chinese medicine (TCM) (Zhou et al., 2018; Xiang et al., 2020). Modern pharmacological research suggests that rhubarb exhibits antitumor, anti-inflammatory, anti-diabetic and neuroprotective effects (Deitersen et al., 2019; Dong et al., 2020; Zhao et al., 2021). Although the genus contains about 60 species, according to the Chinese Pharmacopoeia (2020), rhubarb is defined as the dried rhizomes and roots of *Rheum tanguticum* Maxim.ex Balf., *Rheum palmatum* L. and *Rheum officinale* Baill. (State Pharmacopoeia Committee, 2020). Conversely, the underground parts of *Rheum rhabarbarum* L., *Rheum hotaoense* C.Y.Cheng & T.C.Kao, *Rheum austral* D.Don, *Rheum undulatum* L. and *Rumex japonicus* Houtt. are used as folk medicines under the name Tu-dahuang in China (Yang et al., 2004). Due to the confusion that stems from these different definitions, the rhubarb raw materials available in China are of varying quality. Since the majority of botanical drugs in the consumer market are composed of plant fragments, slices, or even powders, it has frequently been observed that rhubarb products are purposely or unintentionally blended with species that are closely related. Hence, there is a growing need for trustworthy techniques that provide the ability to verify the authenticity of the raw botanical materials and commercial botanical drugs.

Identification of rhubarb is based primarily on morphological traits, microscopic identification, and chemical profiling, which are often affected by plant growth and environmental conditions, causing difficulty in distinguishing rhubarb in processed botanical drugs (Yang et al., 2004; Zhou et al., 2018). As DNA is present in all tissues and unaffected by external or physiological conditions, molecular biology techniques have proven to be effective in the clear identification of various plant species (Sucher and Maria, 2008). DNA barcoding, which entails the sequencing of a specific and standard DNA segment, was originally proposed by Hebert et al. (2003) and is effective when used for species identification. Because of its high diversity and mutation rate, ITS2 has been shown in recent research to be a promising candidate DNA barcode for the identification and classification of many different plant taxa (Zhao et al., 2015; Zhu et al., 2022). The use of ITS2 region is 73.68% effective in identifying species and has been shown to perform best in the characterization of *Bupleurum* L. members. Moreover, *psbA-trnH* can be used as a supplementary site to improve the success rate in *Bupleurum* L. identification (Chao et al., 2014). The chloroplast *matK* region was shown to be effective for use in the identification of rhubarb species (Yang et al., 2004; Zhang et al., 2014). Chen et al. (2022) found that 14 sites containing nucleotide alterations in *matK* and 8 in *rpl32*, which were found to be valuable in the identification of four *Rheum* species (*Rh. tanguticum, Rh. palmatum*, *Rh. officinale* and *Rh*. *undulatum* L. Since DNA barcoding is based on sequencing results, it is an accurate, albeit costly, method.

DNA barcoding and a non-sequencing-based methodology like high-resolution melting (HRM) analysis combine to provide an analytical method that is more dependable, practical, and cost-effective (Chen et al., 2021). Barcode-DNA HRM (Bar-HRM) analysis is noted for its use in the detection of botanical drug adulterants. The identification of species contained in crude drug samples of *Senna alexandrina* Mill. using HRM based on the ITS1 region was attempted initially by Mishra et al. (2018). Furthermore, Xiong et al. (2016) assessed Bar-HRM’s effectiveness at differentiating between *Hyoscyamus niger* L. and its adulterants. Their findings demonstrated that the method could be used to identify adulterants effectively and determine the amount of *H*.* niger* DNA extract in an admixture.

Rhubarb is a valuable botanical drug and health supplement that is traded throughout the world. However, the contents of commercial rhubarb products, such as traditional Chinese patent medicines (TCPMs), are difficult to identify following the multiple processing steps that take place when creating the formulations, causing difficulties for customs inspectors and trade supervisors. It is crucial to authenticate rhubarb quickly and accurately to guarantee drug safety in the global botanical drugs market. There are currently no reports of *Rheum* species identification using the HRM technique. In our study, we aimed to create suitable rhubarb DNA barcodes for creating Bar-HRM markers for the quick identification of rhubarb and its adulterants, as well as the contents of rhubarb-based TCPMs.

## Materials and methods

### Plant materials

Three official *Rheum* species and their adulterants were represented by 63 fresh root, leaf and stem samples gathered from various parts of China, including Gansu, Qinghai, Sichuan, Zhejiang, and Beijing (Table 1). All samples were authenticated by Prof. Dequan Zhang from the College of Life Science and Technology, Southwest University of Science and Technology. All samples were stored at −20°C for subsequent use. In addition, eight batches of TCPMs, comprising 24 samples containing rhubarb, were obtained as tablets, honeyed pills, water pills, concentrated pills, and granules from drug stores (Table 2). The samples of TCPM were ground and stored in a desiccator for further use.

**TABLE 1:** Information of rhubarb species used in our study, and the results of real-time PCR and HRM analysis using a ITS2 mini-barcode.

Chinese name	Species	Origin	Form of sample	Number of samples	Ct	Tm (°C)	HRM confidence (%)
Zhangye dahuang	*Rheum palmatum*	Gansu	Roots	9	14.89 ± 0.14	92.52 ± 0.05	99.34 ± 0.23
Tanggute dahuang	*Rheum tanguticum*	Qinghai	Roots	24	15.25 ± 0.59	92.84 ± 0.04	99.56 ± 0.22
Tanggute dahuang	*Rheum tanguticum*	Dingxi, Gansu	Roots	4	14.65 ± 0.70	92.77 ± 0.09	99.32 ± 0.33
Yaoyong dahuang	*Rheum officinale*	Ya’an, Sichuan	Roots	8	14.73 ± 0.16	92.42 ± 0.04	99.29 ± 0.55
Yaoyong dahuang	*Rheum officinale*	Mianyang, Sichuan	Root, leaf, stem	3	-^a^	-	-
Tu-dahuang	*Rumex japonicus*	Sichuan	Roots	5	14.68 ± 0.61	92.37 ± 0.04	99.48 ± 0.32
Tu-dahuang	*Rumex japonicus*	Zhejiang	Roots	7	15.24 ± 0.72	92.47 ± 0.03	99.57 ± 0.11
Tu-dahuang	*Rumex* sp.	Beijing	Roots	3	13.81 ± 0.04	92.46 ± 0.04	99.55 ± 0.23

a - *Rheum officinale* from Mianyang was not selected for HRM experiment, because the ITS2 sequences of *Rheum officinale* from Mianyang and Ya’an were the same.

**TABLE 2:** Information of the traditional Chinese patent medicines containing rhubarb, and the results of real-time PCR and HRM analysis targeting ITS2 mini-barcode.

Sample	Code	Type	Ingredients on label	GenBank		Real-time PCR				Identification results
				Species	Identity	Ct	Tm (°C)	HRM cluster	HRM confidence (%)	
Sanhuang tablets	1–3	tablets	*Radix et Rhizoma Rhei*, berberine hydrochloride, *Scutellaria* extractum	*Rh. officinale*	KJ543540.1 (100%)	15.82 ± 0.44	92.85 ± 0.03	*Rh. officinale* SC	91.24 ± 0.46	Authentic rhubarb
Sanhuang tablets	4–6	tablets	*Radix et Rhizoma Rhei*, berberine hydrochloride, *Scutellaria* extractum	*Rh. coreanum*	LC457893.1 (100%)	18.47 ± 0.05	92.59 ± 0.05	-^a^	-	Adulterant
Zhichuang tablets	7–9	tablets	*Radix et Rhizoma Rhei*, *Tribulus terrestris L.*, *Mahoniae Caulis*, *Angelica dahurica*, Borneolum, Pig bile	*Rh. officinale*	KJ543540.1 (99.52%)	22.20 ± 0.24	92.85 ± 0.03	*Rh. officinale* SC	94.06 ± 0.73	Authentic rhubarb
Runchang pills	10–12	concentrated pills	*Persicae Semen*, *Notopterygium incisum*, *Radix et Rhizoma Rhei*, *Angelica sinensis*, *Fructus Cannabis*	*Rh. coreanum*	LC457893.1 (99.48%)	25.92 ± 0.40	92.59 ± 0.06	-	-	Adulterant
Jiuzhi dahang pills	13–15	water pills	*Radix et Rhizoma Rhei*	*Rh. tanguticum*	KJ641592.1 (99.52%)	16.88 ± 0.19	92.65 ± 0.04	*Rh. tanguticum* QH	92.15 ± 0.32	Authentic rhubarb
Maren pills	16–18	honeyed pills	*Cannabis fructus*, *Armeniacae semen amarum*, *Radix et Rhizoma Rhei*, *Citrus aurantium L.*, *Magnolia officinalis*, *Paeoniae radix alba*	*Rh. officinale*	KJ543540.1 (100%)	18.75 ± 0.17	92.92 ± 0.00	*Rh. officinale* SC	95.11 ± 0.74	Authentic rhubarb
Yiqing granules	19–21	granules	*Coptis chinensis* Franch., *Radix et Rhizoma Rhei, Scutellaria baicalensis* Georgi	\ ^b^	\	\	\	\	\	\
Dahuang tongchang granules	23–24	granules	*Radix et Rhizoma Rhei* liquid extract	\	\	\	\	\	\	\

^a^ - HRM, confidence is less than 90% and the species cannot be identified.

^b^ \ not applicable.

### DNA extraction and PCR

Fresh rhubarb plant samples were chopped and thoroughly ground in a mortar and pestle, and then 100 mg was taken for DNA extraction using the method of Cui et al. (2006). 2 g of powdered rhubarb TCPMs was taken for DNA extraction by the method of Cheng et al. (2015). The purity and concentration of the DNA was measured using a standard spectrophotometric method at 260 and 280 nm UV using a Nano-100 spectrophotometer (Aosheng Instrument Company, Hangzhou, China). All DNA samples were stored at −20 °C for subsequent analysis after being diluted to 50 ng/μL.

Three candidate barcodes, ITS2, *psbA-trnH* and *rbcL*, were chosen for PCR amplification utilizing universal primers (Supplementary Table S1). A total PCR reaction volume of 25 μL consisted of 50 ng of DNA, 1 μL of each primer (10 μmol/L), and 12.5 μL of 2X SanTaq PCR Mix (Shanghai sangon Biotech Co., Shanghai, China). The reactions were performed with a Thermal Cycler Block 5020 (Thermo Fisher scientific, United States). A 1% agarose gel stained with GoldView II was used to separate the amplified products for 25 min at 120 V and visualized under Tanon-5200 Multi (Tanon, Shanghai, China).

### DNA barcoding analysis

Beijing Tsingke Biotech Co. sequenced the PCR products. Chromas software was used to remove low quality sequences. BLAST tools were used to compare the sequences in GenBank and determine whether they showed high similarity. Then, the obtained DNA sequences, ITS2, *rbcL* and *psbA-trnH*, were compared for sequence lengths, variant sites and guanine-cytosine (GC) contents differences using MEGA 11.0 software. Intraspecific variation and interspecific differences were also calculated using the Kimura 2-parameter (K2P) model. In addition, we downloaded a total of 11 sequences of six *Rheum* species from GenBank. The GenBank accession numbers of the downloaded sequences have been listed in the Supplementary Table S2. The K2P model was utilized in MEGA 11.0 to construct a phylogenetic tree by neighbor-joining (NJ) with bootstrap testing of 1,000 replicates.

### HRM analysis

The TCPMs’ DNA was severely damaged, making it challenging to extract complete ITS2 sequences. To conduct the HRM experiments, mini-barcode primers (ITS2-39F-293R) were designed in conserved regions based on ITS2 sequence using Primer Premier 5 software (Figure 2). The real-time PCR reaction mixtures contained 10 μL of 2× EvaGreen HRM premix (TIANGEN Biotech), 0.4 μL of each primer (10 μmol/L), and 50 ng of DNA. DNA amplification was performed in a Rotor-Gene Q (QIAGEN, Germany) with real-time PCR conditions: 95 °C 10 min, followed by 45 cycles of 95 °C 5 s and 59 °C 10 s. At the conclusion of every cycle, the fluorescence signals were recorded. The temperature was set to increase from 80°C to 97°C at 0.1°C every 2 s. Each sample had three replicates. The melting curves were analyzed by Rotor-Gene Q Series software, and the normalized and differential melting curves were obtained by setting the confidence interval to 90% (Xiong et al., 2016).

## Results

### Identification of rhubarb species using three candidate barcodes

High sequencing efficiency, good PCR amplification, notable inter-species divergences, and low intra-species variance are the ideal characteristics of DNA barcodes (Chao et al., 2014). In our study, the A260/A280 ratios of all DNAs were between 1.78 and 2.04 to satisfy the requirements of subsequent experiments (Supplementary Table S1). Three DNA barcodes, ITS2, *psbA-trnH* and *rbcL*, were amplified from 63 rhubarb individuals, encompassing five Polygonaceae species. The amplified samples were all obtained successfully according to the PCR results (Supplementary Figure S1). The sequencing efficiencies of ITS2, *rbcL*, and *psbA-trnH* were 83.87%, 90.32%, and 66.12%, respectively. The sequence lengths in alignment were 233-384bp for ITS2, 824 bp for *rbcL*, and 311–434 bp for *psbA-trnH* (Supplementary Table S3). After calculations, the GC contents of the *psbA-trnH* and *rbcL* sequences were found to range from 32.26% to 32.80% and 41.38%–41.99%, respectively. Additionally, there were 27 (3.26%) and 96 (21.92%) variant sites in the *psbA-trnH* and *rbcL* sequences, respectively. The ITS2 sequence had 146 variation sites (37.34%) with a range of GC contents 57.26% to 68.67%. Yao et al. (2010) and Liu et al. (2023) showed that the distribution of GC contents on the plant phylogeny was heterogeneous. In our study, ITS2 contained the highest percentage of variable sites, indicating that this sequence was the most suitable for distinguishing rhubarb species.

**FIGURE 1:**
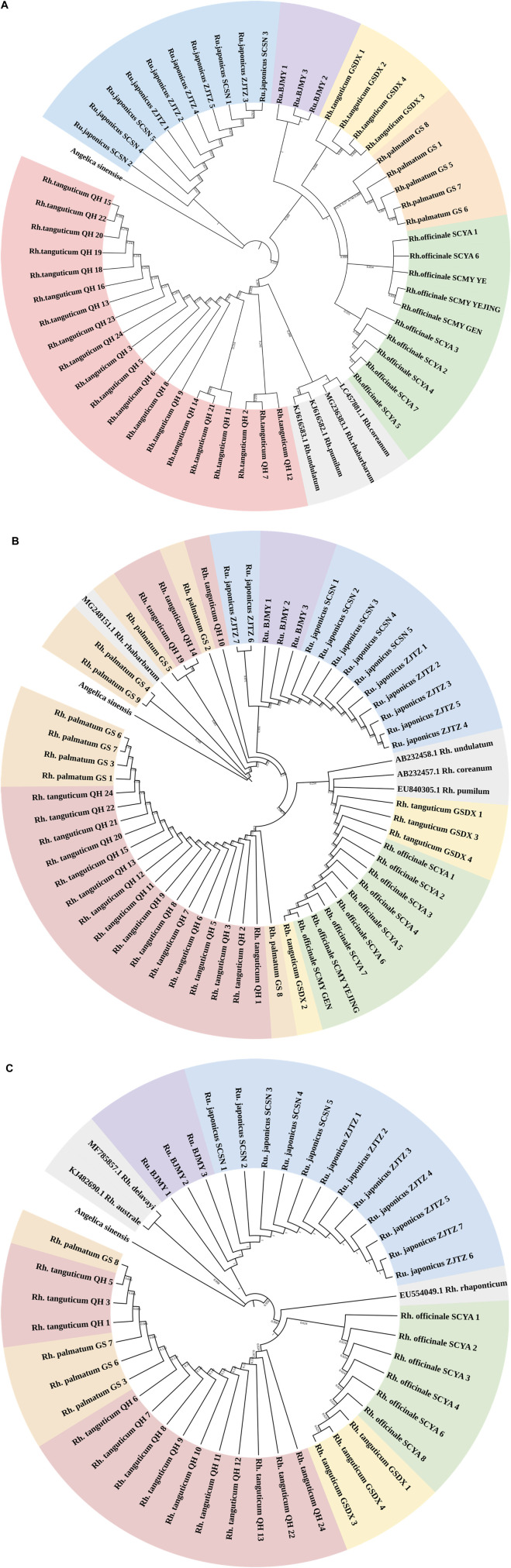
(Continued). NJ tree of Polygonaceae species based on ITS2 **(A)**, *rbcL* **(B)** and *psbA-trnH* **(C)** sequences. Red, yellow, orange, green, blue, purple, and gray regions represent *Rheum tanguticum* from Qinghai, *Rh. tanguticum* from Gansu, *Rh. palmatum* from Gansu, *Rh. officinale* from Sichuan, *Rumex japonicus* from Zhejiang and Sichuan, and *Rumex* sp. from Beijing, and sequences downloaded from GenBank. *Angelica sinensis* as an outgroup.

A suitably broad DNA barcoding gap is necessary for accurate DNA barcoding (Kvist, 2016). The genetic divergence between five Polygonaceae species determined using the K2P model was shown in Supplementary Table S4. The genetic distances revealed a glaring barcoding gap, and the maximum intraspecific genetic distance seen using ITS2 was less than the minimum interspecific genetic distance. As for *rbcL* and *psbA-trnH*, the minimum interspecific genetic distances were lower than the maximum intraspecific genetic distances; thus, the intraspecific and interspecific genetic distances overlapped. Therefore, the *rbcL* and *psbA-trnH* segments were not ideal for use in identifying rhubarb species. We constructed NJ trees of three kinds of sequences using *Angelica sinensis* (Oliv.) Diels as outgroup (Figure 1). Of the three NJ trees, the majority of branches were independent at the species level in the ITS2 topology. Every rhubarb species was placed in a distinct category that did not overlap with any other species. It is worth noting that the ITS2 sequence distinguished *Rh. tanguticum* and *Rumex* from different origins, whereas the *rbcL* and *psbA-trnH* segments could not accurately distinguish all species, and the species classification in the phylogenetic trees was chaotic. Based on an analysis of genetic distances using the NJ tree, it was concluded that ITS2 was the most viable barcode for the identification of the Polygonaceae species.

### Identification of rhubarb and its traditional Chinese patent medicine products using the ITS2 mini-barcode

**FIGURE 2:**
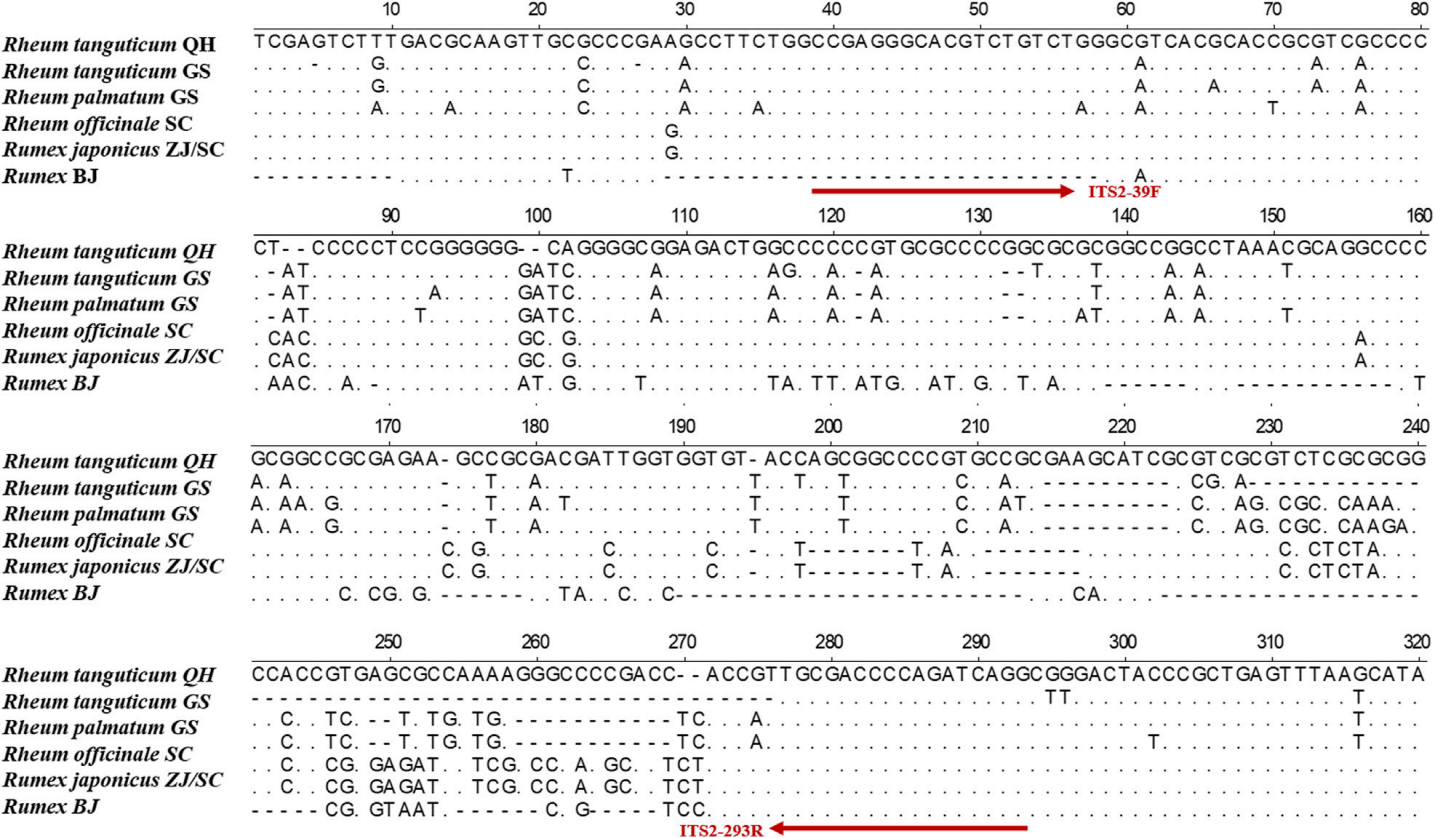
Alignment of ITS2 sequences among *Rh. palmatum*, *Rh. tanguticum*, *Rh. officinale*, *Ru. japonicus*, and *Rumex* sp.

We collected eight batches of TCPMs from drug stores in various geographic areas of China. The DNA of the TCPMs was severely damaged, making it challenging to extract the whole ITS2 sequence. Thus, a variable region of 247 bp was chosen from the ITS2 sequence to produce a mini-barcode. Primers (ITS2-39F-293R) were designed for this region (39–293 bp) of the alignment sequence (Figure 2). ITS2 mini-barcode sequences were amplified successfully in six batches of TCPMs, although not in the remaining batch, Dahuang tongchang (TC) and Yiqing (YQ) granule samples (Supplementary Figure S2). Table 2 showed the all comparisons. The BLAST results showed that tablet samples 1–3, 7–9 and pill samples 16–18 were composed of *Rh. officinale*, and pill samples 13–15 were composed of *Rh. tanguticum*. However, tablet samples 4-6 and pill samples 10–12 were composed of *Rheum coreanum* Nakai, although they were labeled as containing official rhubarb species. These findings showed that the ITS2 mini-barcode could be used to identify TCPMs.

### HRM analysis of fresh rhubarb and traditional Chinese patent medicine samples

**FIGURE 3:**
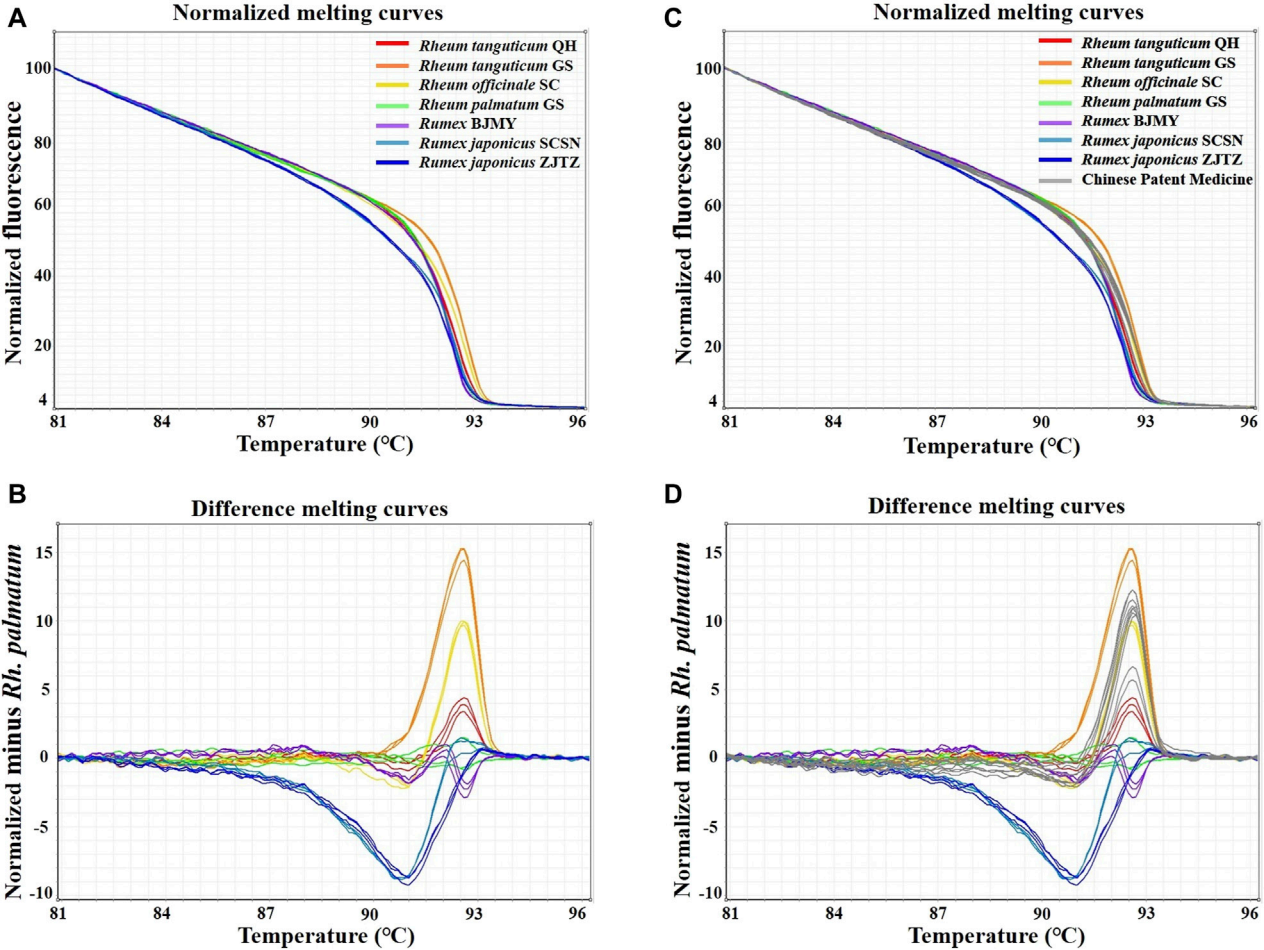
Real-time PCR normalized melting curves and difference melting curves from the HRM analysis of rhubarb species and TCPMs. *Rh. palmatum* was used as the reference genotype in normalized melting curves **(A, B)** and difference melting curves **(C, D)** of all samples. TCPMs include Sanhuang tablets, Zhichuang tablets, Jiuzhi dahang pills, Maren pills and Runchang pills.

In this study, HRM assays were conducted using the primers ITS2-39F-293R, based on ITS2 sequences. Table 1 showed the detailed identification results. A melting curve is deemed to be from a different species if the similarity between the curves each species is less than 90%, according to the 90% genotype confidence percentage (GCP) threshold (Xiong et al., 2016). For the melting curve analysis, *Rh. palmatum* was used as the reference cluster. Obvious disparities were shown in the difference curves of the rhubarb species (Figure 3A, B). The rhubarb samples were sorted into seven clusters that were simple to identify. Of note, *Rh*. *tanguticum* from two origins (Qinghai and Gansu) fell into different clusters. These results implied that HRM using the ITS2 mini-barcode could be used to differentiate rhubarb from its adulterants and identify the origin of each sample.

Furthermore, the primers ITS2-39F-293R were utilized to carry on HRM analysis on eight batches of TCPMs. All TCPMs can have their resemblances to reference genotypes automatically computed by setting the GCP to 90% and allocating the melting curve of every authenticated rhubarb to the reference genotype. The normalized melting curves and difference melting curves were displayed in Figure 3C, D. The specifics of HRM outcomes were shown in Table 2. Apart from the TC and YQ granule samples, 18 TCPM samples underwent effective amplification, with the Ct values ranging from 15.49 to 26.36. In terms of classification of samples into clusters, the tablet samples 1–3, 7–9 and pill samples 16–18 were classified into the *Rh. officinale* SC cluster, while the pill samples 13–15 were grouped into the *Rh. tanguticum* QH cluster. However, tablet samples 4-6 and pill samples 10–12 were not identified as being comprised of official rhubarb species. Based on these findings, 67.7% of the 18 TCPM samples were identified as being composed of official rhubarb, whereas 33.3% of the samples were adulterants or did not contain rhubarb.

## Discussion

As the TCM industry is developing quickly, it is becoming increasingly crucial to have rigorous means to guarantee the efficacy and security of these medicines. However, the components of TCPMs are diverse and the processing methods are complex. Moreover, there are problems with substitution, adulteration, and counterfeiting of these medicines. In China, rhubarb is one of the most widely utilized botanical drugs. In all, the National Medical Products Administration has recorded over nine hundred proprietary TCMs that contain rhubarb (Li et al., 2021). Thus, it is critical to provide a quick, accurate, and dependable way to guarantee TCPM quality.

DNA barcoding has grown in popularity over recent years as a method for the taxonomic identification the plant-based components of botanical drugs, and the technique is crucial to the authenticity of TCMs. Chen et al. (2022) and Zhang et al. (2014) both found that three official rhubarbs and their adulterants could be distinguished by the nucleotide differences in the *matK* sequence. However, in this study, we found that the sequencing results from the *matK* sequence in our samples were poor. Among the remaining three candidate barcodes, *psbA-trnH* and *rbcL* sequences contained few variation sites and had poor ability to distinguish rhubarb species. A number of other studies have revealed that, because the *rbcL* and *psbA-trnH* regions frequently lack variations in related species, they are useful for solving phylogenetic puzzles at higher taxonomic levels but inadequate for species classification (Dong et al., 2012; Chao et al., 2014; Zhu et al., 2022). ITS2 contains a large number of variable sites and a clear barcoding gap to enable official rhubarb to be distinguished from its adulterants. According to our findings, the ITS2 sequence is the ideal choice for rhubarb species identification and shows great potential for origin identification.

In recent years, there has been a big increase in the successful application of the novel and sophisticated technique known as HRM analysis for the identification of TCMs (Li et al., 2018). According to Osathanunkul et al. (2018), Bar-HRM was used to identify adulterants in *Ophiocordyceps sinensis* products available on the market and distinguish between *O*.* sinensis* and *O. militaris* species. Importantly, previous studies have focused only on the original rhubarb species used upstream in the TCM production chain, whereas there have been few reports on the identification of rhubarb in TCPMs downstream of the production chain (Zhou et al., 2017; Asanuma et al., 2019). At present, DNA barcoding and DNA metabarcoding have been used to identify rhubarb TCPMs (Tian et al., 2014; Xin et al., 2022), but HRM has not been used to identify rhubarb TCPMs. The DNA of the TCPMs used in this study was found to be highly degraded. A mini-barcode, using a sequence (100–250 bp) that was substantially shorter than the original barcode length, was developed to increase the PCR amplification success rate in the DNA-degraded samples (Yang et al., 2022).

In this study, the mini-barcode designed according to ITS2 sequences successfully amplified the target fragments from 18 TCPM samples and identified the specific species of rhubarb contained in them. It was notable that 33.3% of the TCPMs contained an adulterant (*Rh. coreanum*), although they were labeled as containing official rhubarb. These findings indicated that ITS2 mini-barcode marker was effective in distinguishing TCPMs containing rhubarb and ensure accuracy in the labeling of the contents. This technique could, therefore, potentially help to prevent consumer fraud and the mislabeling of rhubarb goods. However, HRM also has some limitations. Compared with other new technologies such as DNA metabarcoding, HRM is limited by the deviation generated by PCR amplification process, which is easy to affect the changes of the melting curve. Based on the specificity of primers, only single species sequence information of mixed samples can be analyzed (Zou et al., 2023; Cui and Hou, 2024). In the future, HRM could be combined with other methods such as DNA metabarcoding, microfluidic enrichment barcoding and gold nanoparticles to identify TCPMs.

## Conclusion

In this work, we proposed DNA barcoding combined with HRM as a highly specific method for distinguishing various rhubarb species (*Rh. tanguticum, Rh. palmatum*, *Rh. officinale* and *Rumex*) and the components of 18 TCPM samples. ITS2 was the most appropriate DNA barcode for identifying rhubarb species and tracking the geographic origins of the species. Furthermore, Bar-HRM analysis showed that 33.3% of the 18 TCPM samples were composed of adulterants. To our knowledge, this is the first study using Bar-HRM technology for the simple, fast, and accurate discrimination of rhubarb species and TCPM contents, ensuring the safety of TCPMs and promoting development of the rhubarb industry.

## Data Availability

The 52 ITS2 sequences and 41 psbA-trnH sequences can be found in the NCBI database; accession numbers—PP860965-PP861016 and PP858630-PP858670, respectively. For further queries, please contact the corresponding authors.
