# Supplementary material for: DNA barcoding combined with high-resolution melting analysis to discriminate rhubarb species and its traditional Chinese patent medicines
Source: Front Pharmacol. 2024 Jun 14;15:1371890. doi: 10.3389/fphar.2024.1371890 (PMC11211599; doi:10.3389/fphar.2024.1371890)

**SUPPLEMENTARY MATERIAL**

**TABLE S1|** Oligonucleotide primers for three candidate barcodes and ITS2 mini-barcode gene.

| **DNA Region** | **Primer** | **Sequence (5′ → 3′)** | **Reference** |
| --- | --- | --- | --- |
| ITS2 | ITS2F | ATGCGATACTTGGTGTGAAT | Chen et al., 2010 |
|  | ITS3R | GACGCTTCTCCAGACTACAAT |  |
| *rbcL* | F | TGTGGACCGATGGACTTA | Chen et al., 2010 |
|  | R | AACACCAGGTAGGGAAAC |  |
| *psbA-trnH* | PA | GTTATGCATGAACGTAATGCTC | Chen et al., 2010 |
|  | TH | CGCGCATGGTGGATTCACAATCC |  |
| ITS2-39F-293R | ITS2-39F | CCGAGGGCACGTCTGTCT | This study |
|  | ITS2-293R | CCTGATCTGGGGTCGCAA |  |

**TABLE S2|** Previously registered ITS2, *rbcL* and *psbA-trnH* sequences of *Rheum* species in GenBank.

| **Species** | **Genbank Accession No.** |
| --- | --- |
| *Rheum rhabarbarum* | MG248151.1, MG248151.1, EU554049.1 |
| *Rheum coreanum* | LC457881.1, AB232447.1 |
| *Rheum pumilum* | KJ616582.1, EU840305.1 |
| *Rheum undulatum* | KJ616583.1, AB232458.1 |
| *Rheum delavayi* | MF785857.1 |
| *Rheum australe* | KJ482690.1 |

**TABLE S3|** Characteristics of sequences of three candidate barcodes.

|  | **ITS2** | ***rbcL*** | ***psbA-trnH*** |
| --- | --- | --- | --- |
| **Number of sequences** | 52 | 56 | 41 |
| **Sequence length (bp)** | 233-384 | 824 | 311-434 |
| **Sequence length in alignment (bp)** | 391 | 827 | 438 |
| **Sequencing success rate (%)** | 83.87 | 90.32 | 66.12 |
| **C + G (%)** | 57.26-68.67 | 41.38-41.99 | 32.26-32.80 |
| **Variation sites** **(%)** | 37.34 | 3.26 | 21.92 |
| **Conserved sites (%)** | 62.66 | 96.74 | 78.08 |
| **Parsimony informative sites (%)** | 35.55 | 1.81 | 21.69 |
| **Singleton sites (%)** | 1.79 | 0.24 | 0.23 |

**TABLE S4|** Genetic divergences of five Polygonaceae species using the K2P model.

|  | **Intraspecific genetic**  **divergences** | | | **Interspecific genetic**  **divergences** | |
| --- | --- | --- | --- | --- | --- |
|  | **maximum** | **mean** | **minimum** | | **mean** |
| **ITS2** | 0.0252 | 0.0031±0.0047 | 0.0538 | | 0.1825±0.0764 |
| ***rbcL*** | 0.0050 | 0.0009±0.0016 | 0 | | 0.0082±0.0064 |
| ***psbA-trnH*** | 0.0023 | 0.0000±0.0003 | 0 | | 0.2572±0.1945 |

**FIGURE S1|** PCR amplification of the fresh rhubarb samples with ITS2 (A), *rbcL* (B) and *psbA-trnH* (C) sequences. M: 2000 DNA marker, 1-24: *Rheum tanguticum* QH, 25-28: *Rheum tanguticum* GS, 29-37: *Rheum palmatum* GS, 38-44: *Rheum officinale* SCYA, 45-48: *Rheum officinale* SCMY, 49-55: *Rumex japonicus* ZJTZ, 56-60: *Rumex japonicus* SCSN, 61-63: *Rumex* BJMY.


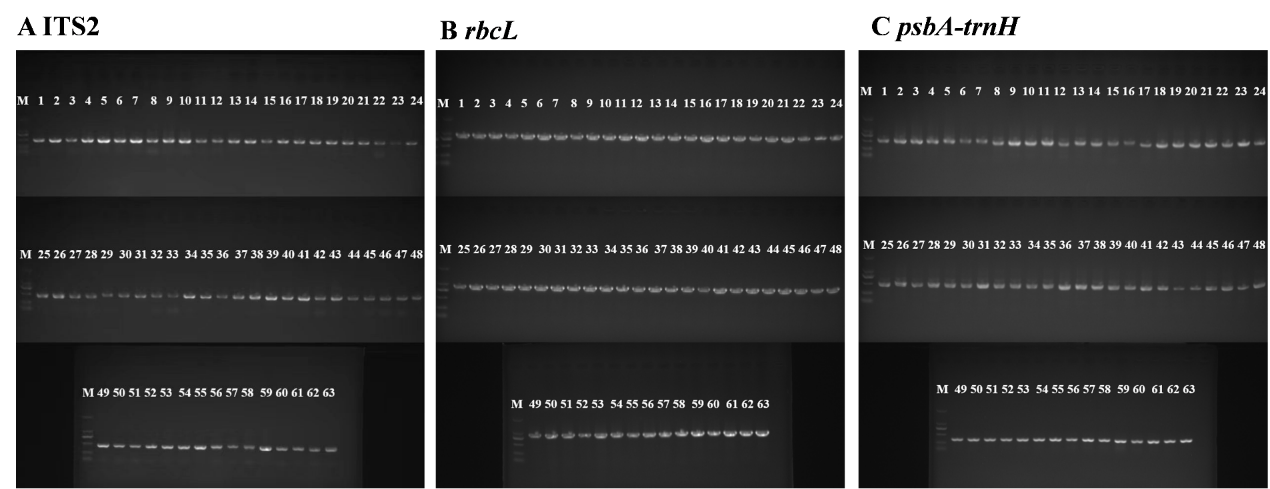


**FIGURE S2|** PCR amplification of the TCPM samples with ITS2-39F-293R primers. M: 2000 DNA marker, 1: Dahuang tongchang granules, 2-3: Sanhuang tablets, 4: Zhichuang tablets, 5: Runchang pills, 6: Jiuzhi dahang pills, 7: Maren pills, and 8: Yiqing granules.


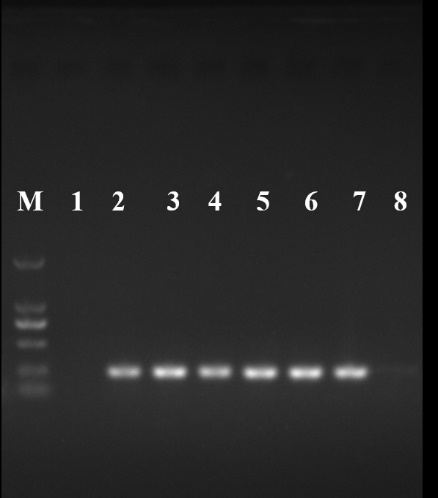

Supplement: Supplementary file 1 [file DataSheet1.docx]
